# Avian Influenza A(H7N9) Virus Infections, Shanghai, China

**DOI:** 10.3201/eid1907.130523

**Published:** 2013-07

**Authors:** Zeng Mei, Shuihua Lu, Xianzheng Wu, Lingyun Shao, Yu Hui, Jiali Wang, Tao Li, Haixia Zhang, Xiaohong Wang, Feifei Yang, Jialin Jin, Ying Zhang, Wenhong Zhang

**Affiliations:** Children’s Hospital of Fudan University, Shanghai, China (Z. Mei, Y. Hui, X. Wang);; Fudan University, Shanghai (S. Lu, T. Li, W. Zhang, Y. Zhang);; Tongji Hospital of Tongji University, Shanghai (X. Wu, H. Zhang);; Huashan Hospital of Fudan University, Shanghai (L. Shao, J. Wang, F. Yang, J. Jin, W. Zhang);; Johns Hopkins University, Baltimore, Maryland, USA (Y. Zhang)

**Keywords:** avian influenza virus, viruses, H7N9, influenza, infections, outbreak, Shanghai, China

**To the Editor:** On March 31, 2013, the National Health and Family Planning Commission of China notified the World Health Organization of 3 cases of human infections with avian influenza A(H7N9) virus. These cases were caused by a novel virus that was identified by laboratory testing at the China Centers for Disease Control and Prevention (CDC) on March 29 (*1*).

As of April 19, 2013, a total of 91 laboratory-confirmed human cases (17 deaths) of infection with avian influenza A(H7N9) virus were reported in 4 provinces in China (*2*). We report clinical features of 2 infected adults who died, 2 critically ill infected adults who recovered, and 1 infected child who had a mild case during this outbreak in Shanghai, China.

A 3.5-year-old boy had fever (39.5°C) for 3 days and mild rhinorrhea starting on March 31. He was admitted to a district pediatric outpatient clinic on April 1. At admission, the child was given oseltamivir for 5 days, even though signs and symptoms had resolved. Nasopharyngeal swab samples were positive by real-time PCR for avian influenza A(H7N9) virus. All symptoms resolved uneventfully by April 3, and CDC was notified that avian influenza A(H7N9) virus was identified in his respiratory sample. The patient was discharged on day 11 after illness onset.

The 4 adult patients were given diagnoses of severe pneumonia with shortness of breath, dyspnea, and marked hypoxia (Table). Duration from disease onset to severe illness was 5–7 days. At admission, the 4 patients with severe cases had decreased peripheral blood leukocyte counts and increased levels of aspartate aminotransferase; 3 had increased levels of lactate dehydrogenase (Table).

**Table:** Characteristics for 4 patients infected with avian influenza A(H7N9) virus, Shanghai, China*

Chacteristic	Patient 1†	Patient 2	Patient 3	Patient 4
Age, y/sex	52/F	49/M	67/M	65/M
Exposure to poultry	None	Continuous	None	None
Sign or symptom at admission	Fever (40.6°C) for 7 d, cough for 1 d, difficulty breathing starting 7 d after illness onset	Fever (39.8°C) for 3 d, cough for 5 d, difficulty breathing and cyanosis starting 5 d after illness onset	Fever (39.7°C) and cough for 7 d starting 7 d after illness onset	Fever (39.0°C) for 5 d, cough for 2 d starting 5 d after illness onset
Physical examination results	HR 120 bpm, RR 40 breaths/min, BP 140/75 mm Hg, decreased breath sounds, no rales	RR 40 breaths/min, BP 240/160 mm Hg, diffuse moist rales	HR 100 bpm, RR 30 breaths/min, BP 110/78 mm Hg, moist rales mainly in left lung	HR 82 bpm, RR 21 breaths/min, BP 118/74 mm Hg, decreased breath sounds in lower left lung, no rales
Laboratory results				
Leukocyte count, ×10^9^/L	3.29	2.9	2.89	3.74
Neutrophils, %	92	69.1	78.6	76.7
Lymphocytes, %	5.5	25.2	15.4	18.2
Platelet count, ×10^9^/L	155	71	172	82
AST, U/L	95	258	45	77
LDH, U/L	525	>2,150	209	492
CPK, U/L	351	>1,600	170	1,854
CK-MB, U/L	16	32	7	31
Creatinine, μmol/L	69.7	116.0	84.2	74.3
Medications after hospitalization				
Oseltamivir	Started d 13 after illness onset	None	Started d 11 after illness onset	Started d 10 after illness onset
Antimicrobial drugs	MOX started d 13 after illness onset	MOX started d 10 after illness onset	AZT started d 11 after illness onset, MOX started d 15 after illness onset	CEF started d 11–12 after illness onset, MOX started d 13 after illness onset
Corticosteroids	MEP, 80 mg/d started d 14 after illness onset	MEP, 80 mg/d started d 10 after illness onset	MEP, 80 mg/d started d 11 after illness onset, decreased to 40 mg/d, stopped after 1 wk	None
Immunoglobulin	Started d 13 after illness onset	None	Given d 11–15 after illness onset	None
Other conditions	Diabetes mellitus, surgery for thyroid cancer	Obesity	None	Hypertension
Outcome	Died 14 d after illness onset	Died 10 d after illness onset	Discharged 30 d after illness onset	Discharged 27 d after illness onset

*HR, heart rate; RR, respiratory rate; BP, blood pressure, AST, aspartate aminotransferase; LDH, lactate dehydrogenase; CPK, creatine phosphokinase; CK-MB, creatine kinase isoenzyme MB; MOX, moxifloxacin; AZT, azithromycin; CEF, ceftriaxone; MEP, methylprednisolone. †Data for patient 1 were reported by Yang et al. (*3*) and are included for comparison.

All 4 adult patients had radiologically confirmed pneumonia and bilateral patchy alveolar opacities or diffused lobar consolidation with or without pleural effusion (Figure, Appendix). Findings on chest radiographs for severe cases requiring mechanical ventilation were consistent with those for acute respiratory distress syndrome.

**Figure:**
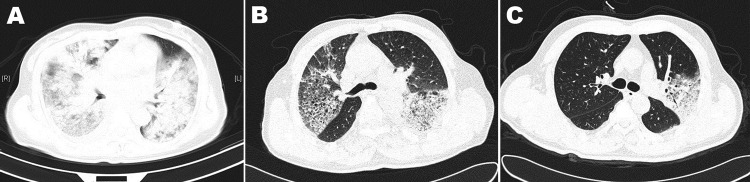
Chest computed tomographic scans for 3 patients infected with avian influenza A(H7N9) virus, Shanghai, China. A) Patient 1, who died, showing extensive lung infiltrates at day 7 of illness onset. B) Patient 3, who had a severe case, showing partial rear lung infiltrations on both sides of the lung and partial normal lung at day 7 of illness onset. C) Patient 4, who had a mild case, showing only partial left lung lobar involvement at day 9 of illness onset.

Among the 4 severe cases in adults, a 52-year-old woman (patient 1) and a 49-year-old man (patient 2) died from acute respiratory distress syndrome and multiple organ failure on days 14 and 10, respectively, after disease onset and 1–2 days after progression to respiratory failure. Two other patients showed improvement and were virus negative 6 and 4 days after antiviral treatment. After 23–24 days of treatment in an intensive care unit, the 2 patients with severe cases recovered and were discharged (Table).

The 2 patients who died were given methylprednisolone. Of the 2 patients who recovered, 1 was given a low dose of methylprednisolone for 1 week and the other was not given methylprednisolone. Although it is difficult to assess the role of glucocorticoids in treatment because of limited number of cases, caution is advised because of possible serious adverse events, including death, as reported for human infection with influenza A(H1N1) virus (*4*).

One of the adult patients reported exposure to poultry. The family of the child patient raised chickens and ducks, but these animals had no apparent disease, and cloacal swab specimens were negative for avian influenza A(H7N9) virus. One patient who died (patient 2) had frequent occupational exposure to poultry. Sixteen contacts of the child and 45 contacts of the 4 adult patients were monitored, and routine virologic sampling was performed. One contact (husband of patient 1) of a patient who died (Table) became febrile and was positive for avian influenza A(H7N9) virus on April 12 (day 24 after disease onset for patient 1); as of the date of this report, he was receiving treatment in an intensive care unit. However, it is difficult to tell if this is a case of human-to-human transmission or if both persons were exposed to infectious poultry. All remaining contacts had no symptoms and were negative for virus by PCR.

Several features of this avian influenza A(H7N9) outbreak are distinct from those of previous avian influenza outbreaks. Human infection with this virus showed a case-fatality rate of 18.7% (17/91), but this rate is not as high as that for avian influenza A(H5N1) virus (case-fatality rate 59%) (*5*).

Avian influenza A(H7N9) virus infection seems to cause more severe human illness than do other subgroups of H7 influenza A viruses (subtypes H7N2, H7N3, and H7N7), which are usually associated with poultry outbreaks but cause mild disease in humans. However, infection with avian influenza A(H7N7) virus resulted in the death of a veterinarian during an outbreak in the Netherlands (*6*). In the 5 patients reported here, avian influenza A(H7N9) virus caused fatal disease in 2 adult patients 52 and 49 years of age, who had other medical conditions. Older age has been reported to confer higher risk for developing more severe influenza-associated outcomes (*7*).

In conclusion, these cases indicated that avian influenza A(H7N9) virus might not be as virulent as avian influenza A(H5N1) virus in humans. Avian influenza A(H7N9) virus does not appear to cause obvious disease in poultry and causes mild disease in children. More severe disease in adults occurred among those had concurrent diseases or were immunodeficient.
